# Mitochondrial and Nuclear DNA Survey of *Zootoca vivipara* across the Eastern Italian Alps: Evolutionary Relationships, Historical Demography and Conservation Implications

**DOI:** 10.1371/journal.pone.0085912

**Published:** 2014-01-17

**Authors:** Luca Cornetti, Michele Menegon, Giovanni Giovine, Benoit Heulin, Cristiano Vernesi

**Affiliations:** 1 Department of Biodiversity and Molecular Ecology - Centre for Research and Innovation, Fondazione Edmund Mach, S. Michele all’Adige, Italy; 2 Department of Life Sciences and Biotechnology, Università di Ferrara, Ferrara, Italy; 3 Museo di Scienze, Trento, Italy; 4 Stazione sperimentale regionale per lo studio e la conservazione degli anfibi in Lombardia, Lago di Endine – Casazza, Bergamo, Italy; 5 Station Biologique, CNRS UMR 6553, Paimpont, France; University of Milan-Bicocca, Italy

## Abstract

The European common lizard *Zootoca vivipara* exhibits reproductive bimodality, with populations being either viviparous or oviparous. In the central-eastern Italian Alps oviparous populations (*Z. v. carniolica*) and viviparous populations (*Z. v. vivipara*) partly overlap geographically. Studying the evolutionary relationship between these taxa presents an interesting opportunity to gain insight into the evolution of this trait. We aim to*:* i) test whether *Z. v. carniolica*, which is endangered, constitutes an ESU (Evolutionary Significant Unity); ii) infer mtDNA divergence time between the *Z. v. carniolica* clade and all the other *Z. vivipara* subspecies with the aid of an external calibration point; and iii) describe the phylogeographical and demographic scenarios in the area. To do so we sequenced about 200 individuals for mitochondrial variation; 64 of them were also analysed for three nuclear genes. Furthermore, we analysed the same nuclear markers in 17 individuals from the other oviparous subspecies *Z. v. louislantzi* and 11 individuals of *Z. v. vivipara* from widespread geographical origins. The mtDNA and nDNA loci that we examined supported the monophyly of *Z. v. carniolica*. The mtDNA-based estimate of divergence time between *Z. v. carniolica* and all the other subspecies indicated a separation at 4.5 Mya (95% CI 6.1–2.6), with about 5% of sequence divergence. Considering that *Z. v. carniolica* harbours higher genetic diversity, while *Z. v. vivipara* from central-eastern Alps shows a signature of recent population and spatial expansion, we argue that *Z. v. carniolica* represents a distinct evolutionary unit, with a presumably long-term evolutionary history of separation. *Z. v. carniolica* populations, occurring at higher latitudes and altitudes than insofar supposed, live in peat bogs, a seriously threatened habitat: taking into account also its evolutionary distinctness, specific conservation measures should be considered.

## Introduction

The Eurasian common lizard, *Zootoca vivipara* (Jacquin, 1787), is among the few squamate reptiles displaying reproductive bimodality at the intraspecific level.

Viviparous (or better ‘lecithotrophic viviparity’, i.e. live-bearing with nutrition from the yolk, [1]) populations, ascribed to the nominotypical subspecies *Zootoca vivipara vivipara*, are widely distributed from the British Isles and central France to Scandinavia and north-eastern Asia as far as Japan [2]. Oviparous populations are restricted to the southern edges of the range, in two disjunct areas: southern France-northern Spain and northern Italy-southern Austria-Slovenia-Croatia. The French-Spanish oviparous populations have been recently attributed to the subspecies *Z. v. louislantzi* [3], whose range is clearly geographically separated from viviparous *Z. v. vivipara* populations [3]–[6]. All the other oviparous populations are included in the subspecies *Z. v. carniolica* [13]: in this case, the range has been described as parapatric to *Z. v. vivipara* [7]–[9] (Figure S1). However, a contact zone between *Z. v. vivipara* and *Z. v. carniolica* has been recently found in Carinthia, Austria [10].

Using karyotype [11] and mitochondrial DNA (mtDNA) sequence variation [7], [12]–[13], several studies have addressed the phylogenetic relationships between the different subspecies. The scenario can be summarised as follows: the two oviparous subspecies, *Z. v. louislantzi* and *Z. v. carniolica* are not reciprocally monophyletic. Considering the most comprehensive mtDNA survey [13], it appears that *Z. v. carniolica* is sister to all the other subspecies, namely *Z. v. vivipara*, *Z. v. louislantzi* and *Z. v. pannonica* ([14]: in this study the term used was still the former, *Lacerta vivipara pannonica*). Therefore, neither the oviparous (*Z. v. carniolica* and *Z. v. louislantzi*) nor viviparous subspecies (*Z. v. vivipara* and *Z.v. pannonica*) form monophyletic groups, making a single transition from oviparity to viviparity unlikely.

*Z. v. carniolica* has been considered as an Evolutionarily Significant Unit (ESU) *sensu* Moritz (1994) [15]. However, none of the aforementioned studies included nuclear DNA sequence variation. Proving the ESU status for this subspecies would significantly support the conclusions of earlier studies pointing out that *Z. v. carniolica* would deserve specific conservation measures [9].

*Z. v. carniolica* has been found from Piedmont via northern Italy and Austrian Carnian Alps to Slovenia and north-west Croatia, and its northern limit appears to correspond with the Italian Prealps, while the southern limit is represented by a few wetland areas in the Po Valley. The high degree of fragmentation of these low-mid-altitude wetland areas, affected by both climate change and human activities, might pose a serious threat to *Z. v. carniolica* persistence, as highlighted by some local extinction documented in the Po Valley area [16], [17].

The main aim of this work was to test the ESU hypothesis for *Z. v. carniolica* by contrasting patterns of DNA sequence variation at nuclear and mitochondrial markers in 92 and 230 individuals, respectively, of *Z. vivipara* spp. from different European regions. Moreover, we specifically focused on the evolutionary history of *Z. v. carniolica* at both macro- and micro-scale by: a) inferring mtDNA divergence time between the *Z. v. carniolica* clade and all the other *Z. vivipara* subspecies with the aid of an external calibration point; b) describing the phylogeographical and demographic scenarios in an area of partial distribution overlap - central-eastern Italian Alps- between the oviparous populations of *Z. v. carniolica* and the viviparous populations of *Z. v. vivipara*.

## Materials and Methods

### Ethics Statement

All conducted experiments complied with the current laws of Italy. The Italian Ministry of Environment and the Environmental Unit of the Autonomous Province of Trento approved capture, handling, and tissue sampling (DPN/2D/2003/2267 and 4940-57/B-09-U265-LS-fd). In this study we did not apply laboratory techniques on living animals, therefore authorization from the Italian Ministry of Health was not required.

### Sampling

**Figure 1:**
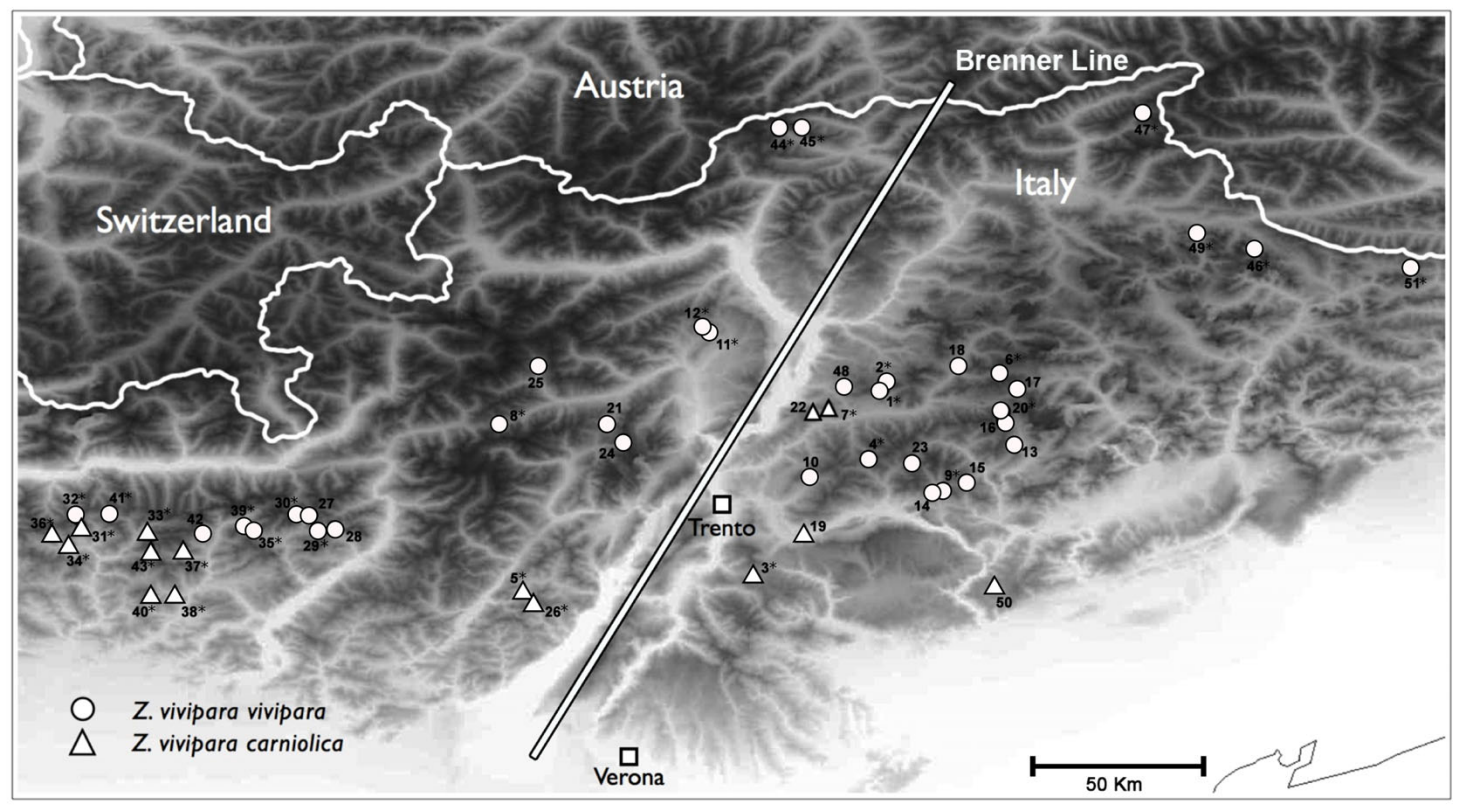
Sampling sites of *Zootoca vivipara* sp. in Northern Italy. Label, site names and coordinates are listed in Table S1a. Circles and triangles indicate locations where we found *Z. v. vivipara* and *Z. v. carniolica,* respectively, according to cytb results. The white line represents the Brenner line. Asterisks indicate locations for which at least one sample was analysed for nuclear genes.

Approximately 1 cm of tail was collected from 191 specimens of *Z. vivipara* coming from 51 locations throughout the central eastern Alps and Prealps (Figure 1 and Table S1a). All animals were released in their own habitat after pouring liquid sterilizer on the tail. Tissue samples were preserved in 95% ethanol and then stored at –80°C until molecular analyses were performed. To have a good representation of the whole geographic distribution and of the known subspecies within the *Zootoca* genus, 39 additional specimens were included in the nuclear marker analyses: their geographical origin and the subspecies they belong to is reported in Table S1b along with their mtDNA haplogroup (previously determined in [13], [18]).

### DNA Extraction, Amplification and Sequencing

DNA was extracted with the commercial QIAGEN DNeasy Tissue Kit (QIAGEN Inc., Hilden, Germany) according to manufacturer’s protocol. A 385 base pair (bp) fragment of mtDNA cytb gene was amplified using MVZ04 and MVZ05 primers [19]. The PCR amplification was carried out in a 20 µl reaction mix containing: 1 µl template DNA, HotMaster™ Taq Buffer 25 mM Mg^2^ (Eppendorf), 100 µM dNTPs, 10 µM of each primer, 0.5 mg/ml BSA and 1 unit of HotMaster™ Taq. The thermocycling regime consisted of incubation at 94°C for 10 min, followed by 35 cycles of 94°C for 1 min, 59°C for 45 s, and 65°C for 1 min, with a final extension of 65°C for 10 min. Moreover, three nuclear genes were investigated. A 572 bp fragment of oocyte maturation factor (C-mos) coding gene, a 447 bp fragment of acetylcholinergic receptor M4 (ACM4) gene and a portion of 579 bp of melanocortin receptor 1 (Mc1r) gene were amplified. All these nuclear sequences have been already used as phylogenetic markers in lacertid species [20], [21]. The amplification protocol consisted of an initial denaturation step at 94°C for 10 min, followed by 35 cycles of the series: 94°C for 1 min, annealing temperature (57°C for Hcmos3 and L-1zmos (C-mos, [20]); 59°C for MC1RF and MC1RR (Mc1r, [22]); 59°C for tg-F and tg-R (ACM4, [23]) for 45 s and 65°C for 1 min; then, a final extension step at 65°C for 10 min. For all amplifications, contamination was rigorously checked by means of blank samples in both extraction and PCR. Before sequencing, the excess primers and dNTPs were removed using ExoSAP-IT (USB Corporation, Cleveland, OH). Sequencing of double-stranded DNA was performed in both directions using a Big Dye Terminator cycle sequencing kit (Applied Biosystems) following manufacturer’s instructions; the sequencing reaction products were run on an ABI Prism 310 Genetic Analyzer (Applied Biosystems). The resulting sequences were edited with FinchTV version 1.4.0 (open source application developed by Geospiza Research Team), sequence fragments were assembled using Sequencher version 4.7 (Gene Codes. Corporation, USA), aligned using Clustal X [24], and checked by eye. All sequences have been deposited in GenBank database under Accession No. KF886538–KF886566.

### Phylogenetic Analysis and Estimation of Divergence Time

We inferred phylogenetic relationships and divergence-times using a relaxed Bayesian molecular clock with an Uncorrelated Lognormal model (BEAST version 1.6; [25]) on a cytb dataset comprising 96 unique haplotypes (*Z. vivipara* of our study plus deposited sequences of *Z. vivipara* spp., *Podarcis peloponnesiaca, Podarcis cretensis* and *Lacerta viridis* as outgroup (GenBank accession numbers: *Z. vivipara*: AY714882–AY714929, *Podarcis peloponnesiaca:* AY896117–AY896123, *Podarcis cretensis*: AF486191–AF4861220 and *Lacerta viridis*: EU116514). JModelTest version 1.0.1 [26] was used to select the appropriate model of evolution for cytb gene under the Akaike Information Criterion AIC [27]. The GTR model of nucleotide substitution with gamma rate heterogeneity among sites and, as a prior, a Yule pure birth model of speciation to estimate the time of divergence between *Z. v. carniolica* and all the others *Z. vivipara* subspecies were used. The analysis was calibrated by setting an age prior on a single node: the divergence of *Podarcis peloponnesiaca* from *Podarcis cretensis* [28]. This divergence can be approximately posed during the Messinian geological events that occurred in Mediterranean Sea at around 5.2+− 0.1 Mya, when Crete became isolated from Peloponnese [29]. We adopted the vicariant event as the most likely explanation for biogeography of Mediterranean Isles as outlined by a previous study [30]. Posterior distributions for each parameter were obtained using a Monte Carlo Markov Chain (MCMC), which was run for 100 million generations, and sampled every 10000 generations. Inspection of the results using Tracer version 1.5 [31] confirmed that stationarity was achieved in all cases and that effective sample sizes (ESS) were adequate (all higher than 200). Trees were summarized as maximum clade credibility trees using the TreeAnnotator program which forms part of the BEAST package, and visualized using FigTree version 1.3.1 (http://tree.bio.ed.ac.uk/software/figtree). In each case, the first 10% of samples was discarded to avoid sampling the burn-in phase. A Bayesian Skyline Plot was also constructed with the software BEAST version 1.6 [25] using the GTR+G evolutionary model, a log-normal relaxed molecular clock with a mean substitution rate of 7.8×10e-9 per site per year and visualized with Tracer version 1.5 [25]. The evolutionary rate was calculated on the basis of the *P. peloponnesiaca* and *P. cretensis* divergence time. This analysis was run multiple times to check for convergence with 50 million iterations and samples drawn every 5000 MCMC steps, after a discarded burn-in of 5 million steps.

To confirm BEAST results and to get Bayesian posterior probability values of the tree we also applied MrBayes version 3.1.2 [32]. We ran the same dataset with 10 million generations (after this number of generations the standard deviation of split frequencies had reduced to less than 1%) with a sampling frequency of 1000, to be sure that a good sample of the posterior distribution had been obtained. The first 2500 sampled trees were discarded as ‘burn-in’ and posterior probabilities were calculated and reported on a 50% majority rule consensus tree of the remaining 7501 trees in the sample. The GTR+G evolutionary model was used. Moreover, a Maximum Likelihood analysis was performed with PAUP* version 4.0 [33] using tree-bisection-reconnection (TBR) branch swapping with 1000 rearrangements and 100 bootstrap replicates.

Additionally, we performed phylogenetic analyses based on three nuclear genes (C-mos, ACM4, Mc1r) on a subset of 92 samples of the entire dataset (64 samples from eastern Alps and 28 from the whole distributional range of the species), selected to include all the major cytb mtDNA clades. We performed phylogenetic tree reconstructions for each single nuclear gene and for a concatenated sequence of 1598 bp. We first applied PartitionFinder version 1.1.1 [34] in order to test the best partition scheme for codon positions and different single gene models of molecular evolution using the Bayesian information criterion (BIC). Four partitions were identified in nuclear sequences: ACM4 and C-mos 1^st^ position (GTR+I+G), C-mos 2^nd^ position (JC), C-mos 3^rd^ position and Mc1r 3^rd^ position (GTR) and Mc1r 1^st^ and 2^nd^ positions (HKY+I). These partitions and models were applied for performing phylogenetic reconstruction for each single nuclear gene and for the concatenated sequence using MrBayes version 3.1.2 [32] for Bayesian analyses with the same settings as for mtDNA; for Maximum Likelihood analyses we used RAxML (version 7.4.2, [35]) and each partition was run under GTR+G model. For nuclear phylogenetic reconstructions, we used *Atlantolacerta andreanskyi* as outgroup (accession numbers: JX485363, JX462052, JX461870), being the phylogenetically closest lacertid lizard with all three nuclear genes available [21]. We chosen not to concatenate mtDNA and nuclear genes, since we focused cytb analysis on the estimation of divergence time between *Z. v. carniolica* and *Z. v. vivipara* taking advantage of a calibrated external node dated on the divergence time between *P. peloponnesiaca* and *P. cretensis*. The three nuclear genes we analysed were not available for the latter species, therefore we ran nuclear analyses separately.

Using the median-joining algorithm in the Network version 4.5.1.0 software [36] we inferred a cytb mtDNA haplotype network, combining our sequences with all those already deposited in public repositories. After phasing nuclear genes with PHASE version 2.1 [37], Network version 4.5.1.0 [36] was also used to obtain haplotype networks of the three nuclear genes. Net nucleotide divergence (Da [38]), defined as distance between cytb clades, was calculated with MEGA version 4 [39]. Standard and molecular diversity indices, neutrality tests and mismatch distribution were calculated using ARLEQUIN version 3.11 [40]. Specific analyses on C-mos sequences for estimating the ratio, ω, between the rate of non-synonymous, dN, and synonymous, dS, substitutions were performed with DNAsp version 5 [41]. The gametic phase of nuclear markers was not considered in phylogenetic analyses.

## Results

### Cytb

**Figure 2:**
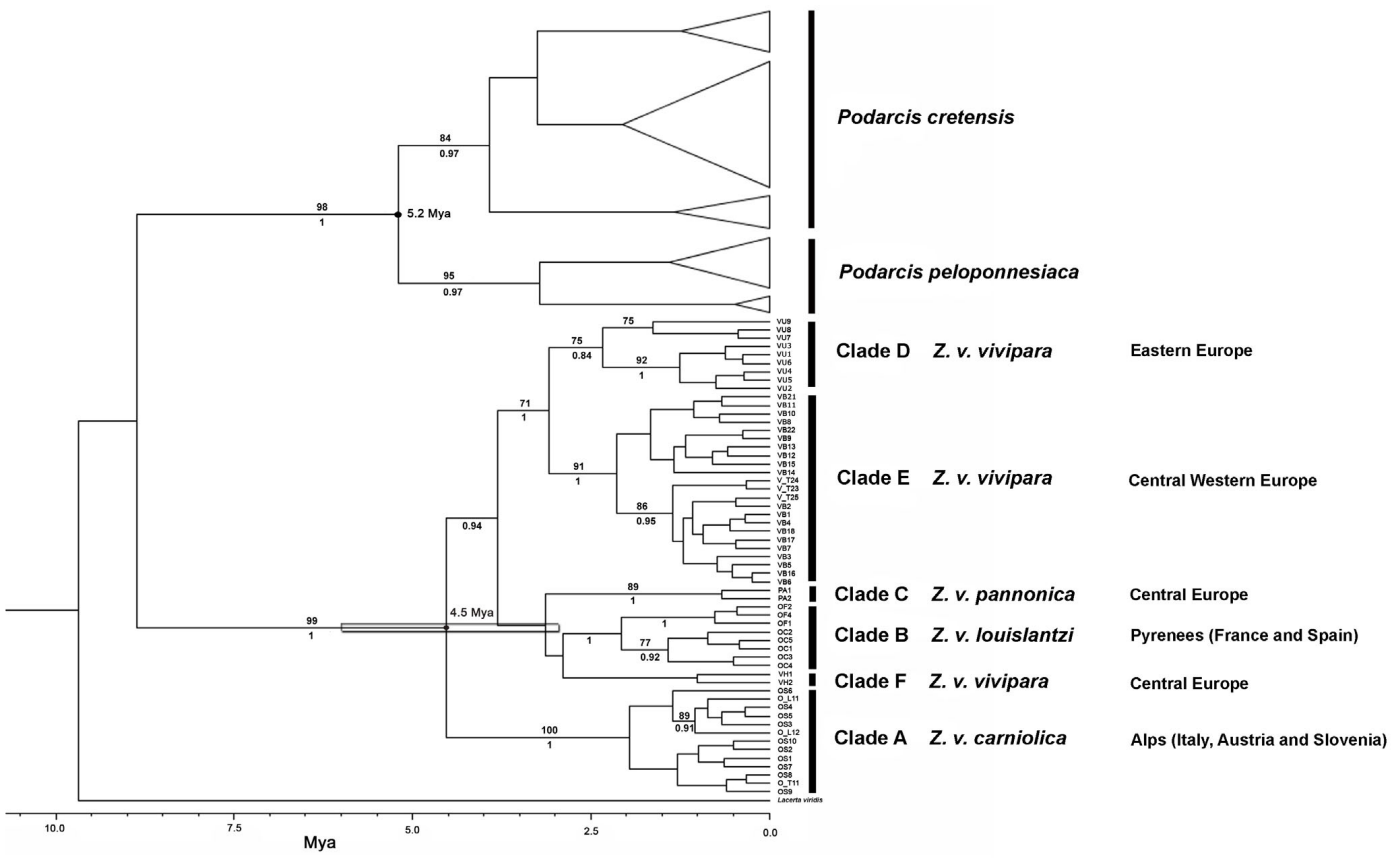
Maximum clade credibility tree from Bayesian analysis of mitochondrial cyt b with chronogram. Bar around the 4.5*Z. vivipara* sp. vs *Z. v. carniolica* shows the 95% Credibility Interval. Bootstrap support values of Maximum Likelihood analysis >70% are shown above the branches, while posterior probability values of Bayesian Inference >0.7 are shown below the branches. Clade names as in Surget-Groba et al. (2002).

A 385 bp portion of the mtDNA cytb gene was examined in our 191 Italian samples. A total of 28 polymorphic sites, all of which were parsimony-informative, and 11 haplotypes were identified. Five of these were new haplotypes, since no match was found with any previous published haplotype (Table S2). No deletions or insertions were observed in our dataset. Mean nucleotide percentage composition was T, 35.5; C, 25.3; A, 26.7; G, 12.4; the estimated transition/transversion ratio was 8.36. We reconstructed a phylogenetic tree using Italian samples from this study and deposited sequences from different clades and origins. The phylogenetic tree obtained using BEAST, ML and Bayesian inference (Figure 2), with *Lacerta viridis* as the outgroup, was topologically similar to others reported in the literature [12], [13]. Indeed, analyses showed clear separation between the clade A, including only *Z. v. carniolica* haplotypes and the remaining clades B, C, D, E and F, comprising all the other European *Z. vivipara* subspecies, which was well supported by bootstrap and posterior probability values. All Italian specimens clustered in two distinct clades: 42 were grouped in clade A, while 149 were placed in clade E, the western European viviparous clade in which only *Z. v. vivipara* individuals have been insofar included.

Estimation of the divergence time between the *Z. v. carniolica* clade A and the clades (B, C, D, E and F) comprising all the other subspecies, namely *Z. v. vivipara*, *Z v. louislantzi*, and *Z. v. pannonica* was obtained by adding a prior of 5.2+- 0.1 Mya on the node separating *P. cretensis* from *P. peloponnessiaca.* Using this calibration, the divergence time between A and all the other clades was found to be 4.5 Mya with a 95% credibility interval between 6.1 and 2.6 Mya (Figure 2).

**Figure 3:**
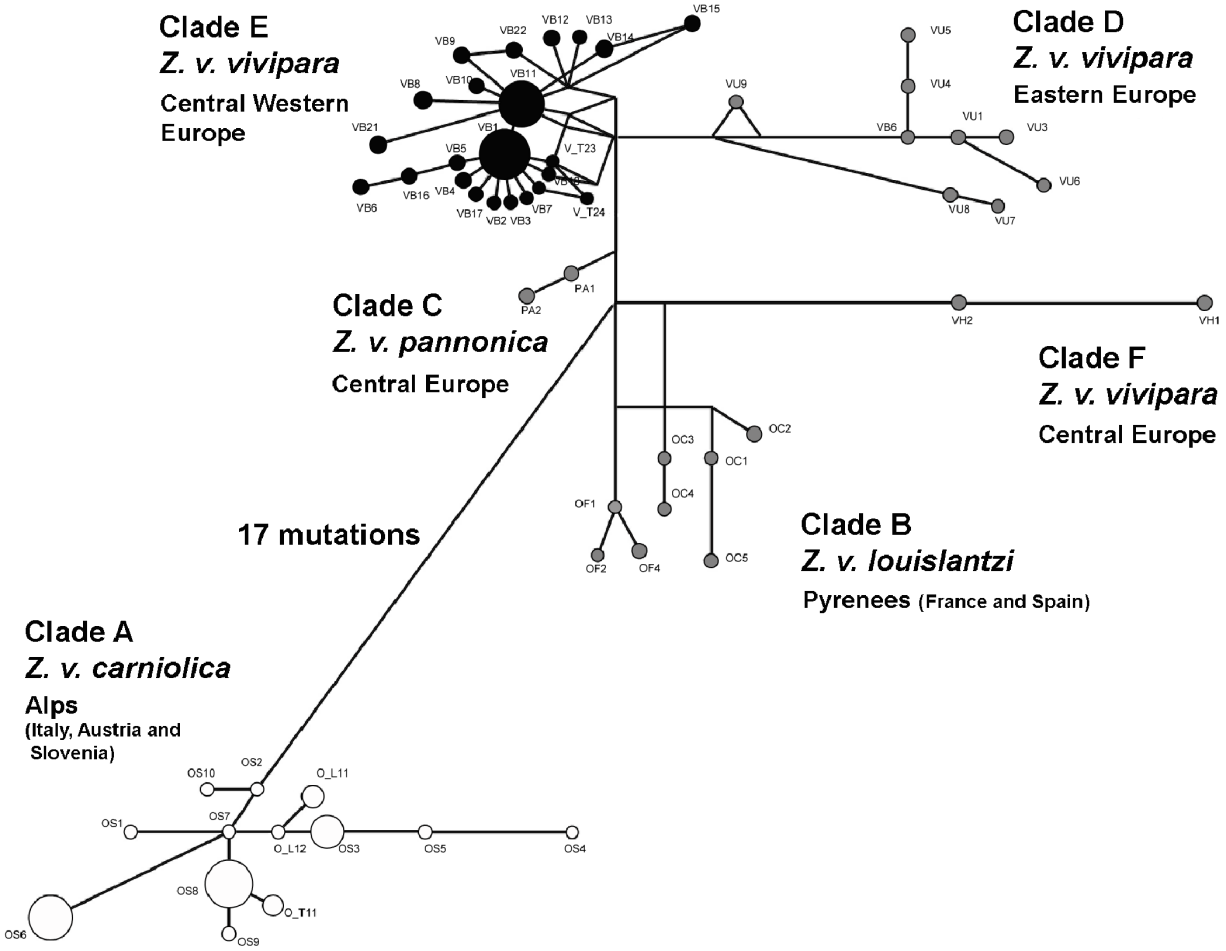
Median joining network of mtDNA cty b haplotypes. Circles represent haplotypes, area is proportional to frequency and colour indicates the subspecies (black, *Z. v. vivipara*; white, *Z. v. carniolica*; grey, other subspecies or European populations).

**Figure 4:**
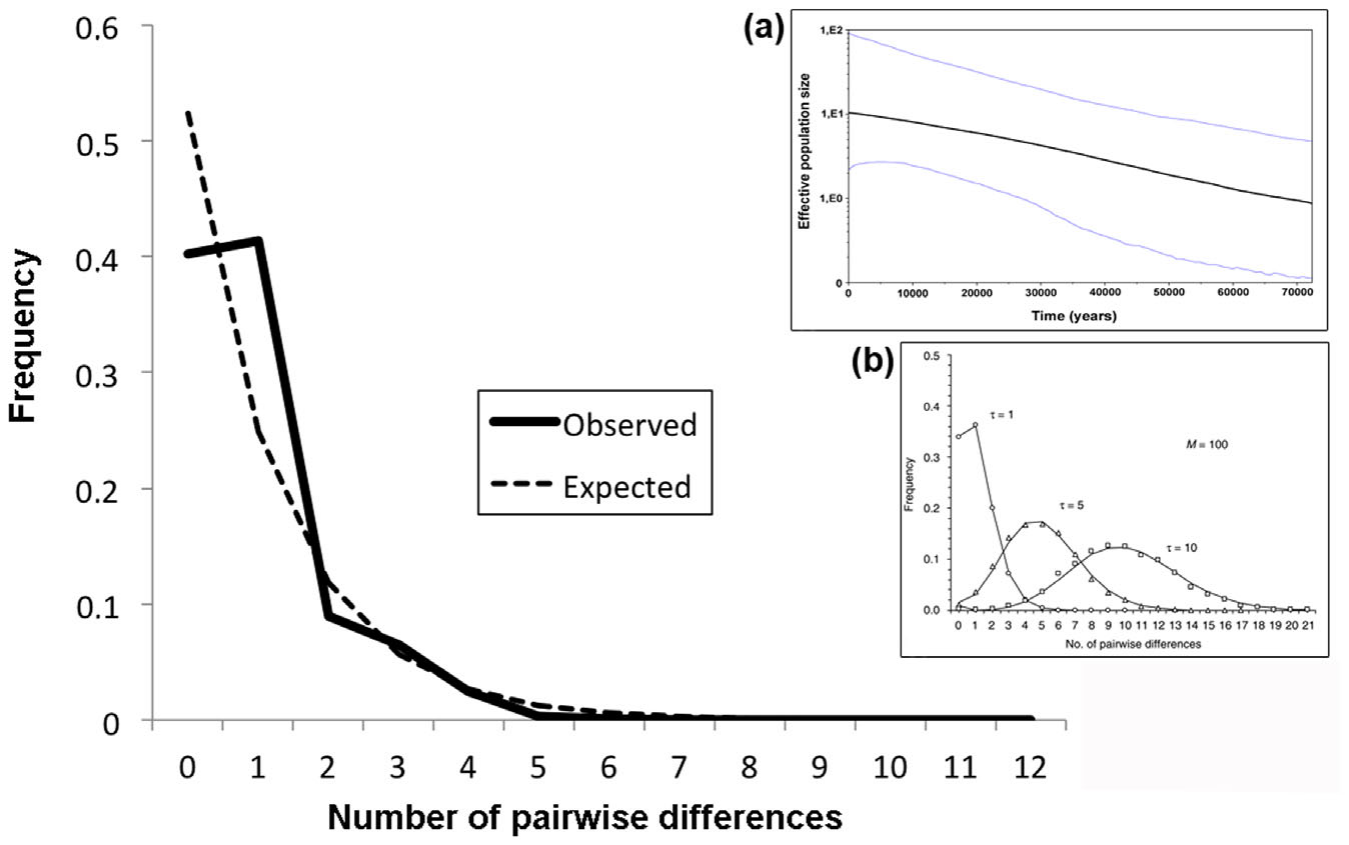
*Z. v. vivipara* (Clade E) cyt b mismatch distribution. The number of nucleotide site differences between pair of individuals and the frequency of observation, are reported on the x- and y-axis respectively. Dashed and thick lines represent observed and expected (under sudden expansion model) distribution, respectively. In the inset: a) Bayesian skyline plot with median value and 95% Credibility Interval; b) mismatch distribution under demographic and spatial expansion model as in Excoffier (2004).

The median joining network (Figure 3) confirmed the clear separation in different clades. As before, 42 individuals of our data set were grouped into clade A, formed by *Z. v. carniolica* haplotypes from Slovenia, Italy and southern Austria [13], while the other 149 were included in clade E, consisting of haplotypes of *Z. v. vivipara* from northern and western Europe. This viviparous clade E showed a distinctive “star-shape” topology, suggesting that populations of this clade might have experienced a recent demographic expansion. Assuming neutrality, population expansion gives rise to an increase in the number of rarer haplotypes in the population under examination (star-shaped network), which also leads to a unimodal mismatch distribution. To further confirm this demographic scenario, we calculated the mismatch distribution (Figure 4) and the values of the D [42] and Fs [43] statistics, both of which were significantly negative: Tajima’s D = −2.207 and Fs = −24.700 (p<0.01). In the absence of selection, significantly negative values for both statistics are usually interpreted as a signature of population expansion events. Moreover, a confirmation of this demographic expansion was gained through the Bayesian coalescent-based skyline plot (Figure 4, inset a), showing a clear pattern of effective population size (Ne) increase in the last tens thousands years. In contrast to clade E, clade A (corresponding to subspecies *Z. v. carniolica*) did not present a star-shape topology (Figure 3) and both Tajima’s D and Fu’s Fs were not significantly different than 0 (data not shown), thus not showing any departure from neutrality. In addition, clade A showed a multimodal mismatch distribution (Figure S4) and no evidence of expansion through Bayesian skyline plot (Figure S4, inset a).

Finally, we estimated the average number of nucleotide differences between the two groups (clade A and clade E) in which all our Italian individuals were divided: it was equal to 20.801+/−3.974 (SD), hence 0.054 per site. The number of net nucleotide substitutions per site between groups, Da, was 0.049, indicating an average difference of about 5%. The two groups did not share any substitution. The results, reported in Table 1, indicate that despite the lower number of individuals analysed, there was much higher genetic variation within *Z. v. carniolica* clade than in *Z. v. vivipara* clade E.

**Table 1:** Indices of genetic variability for the two subspecies at cyt b and nuclear markers.

Marker	Subspecies	n^a^	k^b^	s^c^	n transitions	n transvertions	π^d^	MPD^e^	H^f^
mtDNA cyt b	*Z. v. vivipara*	149	5	3	3	–	0.001±0.001	0.533±0.434	0.480±0.029
mtDNA cyt b	*Z. v. carniolica*	42	6	9	9	–	0.008±0.005	3.120±1.673	0.721±0.044
nuDNA C-mos	*Z. v. vivipara*	33	1	–	–	–	–	–	–
nuDNA C-mos	*Z. v. carniolica*	31	5	4	2	2	0.002±0.002	1.361±0.860	0.569±0.080
nuDNA Mc1r	*Z. v. vivipara*	33	6	6	5	1	0.003±0.002	2.000±1.240	0.889±0.091
nuDNA Mc1r	*Z. v. carniolica*	31	5	4	4	–	0.002±0.001	1.036±0.745	0.709±0.136
nuDNA ACM4	*Z. v vivipara*	33	3	2	1	1	0.003±0.002	1.333±0.910	0.667±0.131
nuDNA ACM4	*Z. v. carniolica*	31	2	1	1	–	0.001±0.001	0.436±0.421	0.436±0.133

^a^ n, sample size,

^b^ k, number of haplotypes,

^c^ s, number of polymorphic sites,

^d^π, nucleotide diversity,

e MPD, mean pairwise differences,

f H, gene diversity.

### Nuclear Genes

We successfully analysed 92 samples (30 individuals belonging to clade A, 17 to clade B, 4 to clade D, 39 to clade E and 2 to clade F) with three different nuclear genes: C-mos, ACM4 and Mc1r.

In some Lacertid species the presence of several functional and non-functional copies of the C-mos gene has been reported [44]. Before any phylogenetic analysis, it is therefore important to verify that only orthologous C-mos sequences are used for comparisons. None of our sequences presented deletion, insertion or internal stop codons. The ratio ω (dN/dS) was significantly higher than 1, thus rejecting the hypothesis of neutrally evolving sequences, as expected in case of non-functional copies. We concluded that all our C-mos sequences were functional copies of the C-mos gene, being therefore orthologous.

The median joining network of the phased alleles of C-mos and Mc1r, showed that individuals belonging to mtDNA clade A (*Z. v. carniolica*) do not share any allele with individuals from other clades (Figure S2). In contrast, two out of five ACM4 alleles (ACM4_4 and ACM4_6) are shared among individuals of *Z. v. carniolica* and individuals of clades B (*Z. v. louislantzi*), D (*Z. v. vivipara*), E (*Z. v. vivipara*) and F (*Z. v. vivipara*). Similarly, phylogenetic trees obtained from each single nuclear gene suggested a highly supported (bootstrap and Bayesian posterior probability higher than 97% and 0.97, respectively) monophyly of *Z. v. carniolica* in C-mos and Mc1r (Figure S3), but not in ACM4. Phased alleles along with their accession numbers were listed in Table S2.

**Figure 5:**
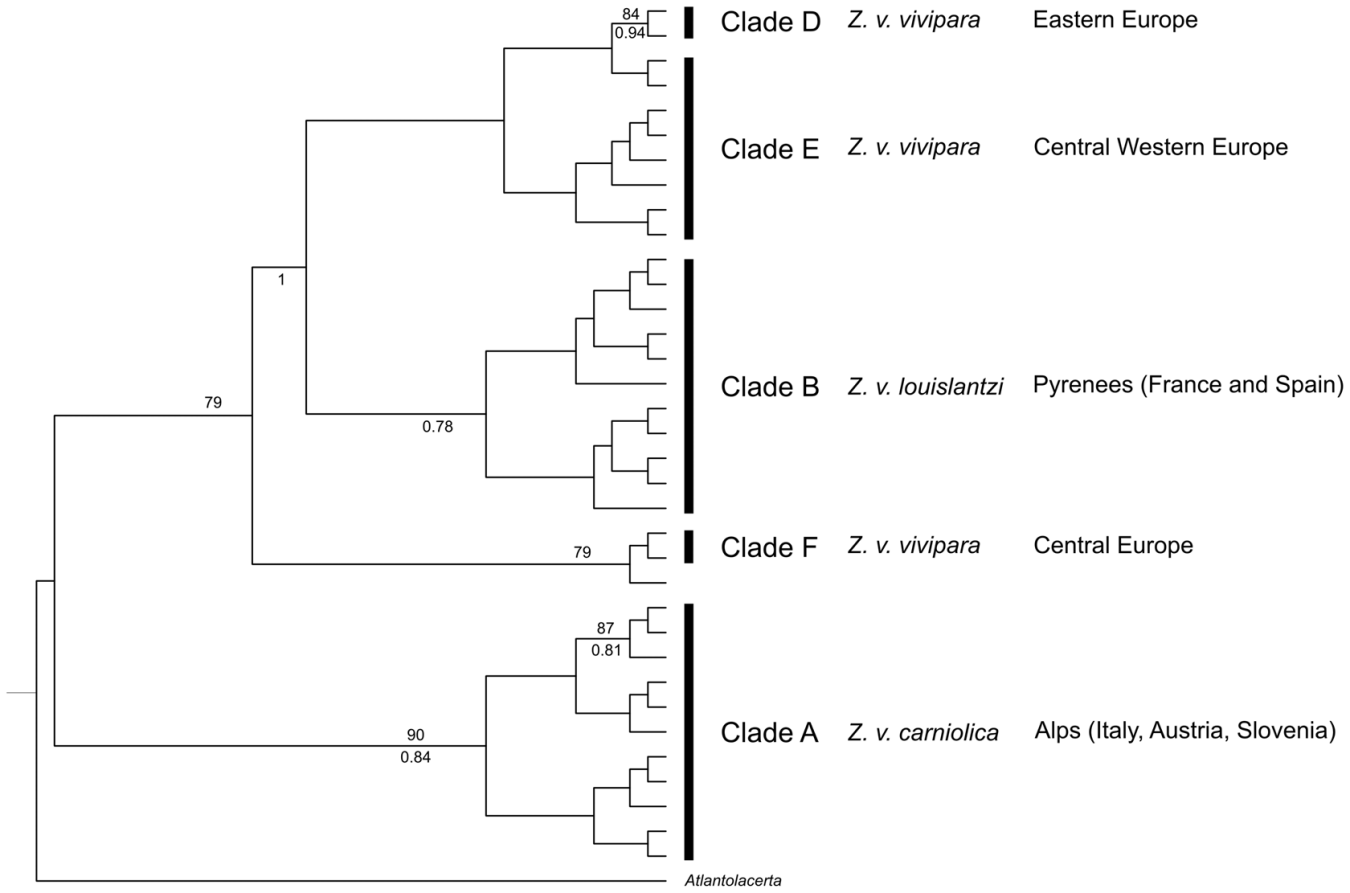
Maximum clade credibility tree of Bayesian analysis of three concatenated nuclear genes variants (C-mos, ACM4 and Mc1r). Bootstrap support values of Maximum Likelihood analysis >70% are shown above the branches, while posterior probability values of Bayesian Inference >0.7 are shown below the branches. *Atlantolacerta andreanskyi* was used as outgroup.

After concatenating the three genes, we obtained a 1598 bp sequence. Thirty-two variants out of the 92 sequences were identified. The tree topology obtained with different methods was concordant so that only the Maximum Clade Credibility Tree is presented in Figure 5. The tree showed two well supported clades. The first composed only of individuals belonging to mtDNA clade A, that is the *Z. v. carniolica* clade (90% and 0.84, bootstrap and Bayesian posterior probability, respectively), whilst the second consisted of individuals with mtDNA belonging to all the other clades, namely B (*Z. v. louislantzi*), D (*Z. v. vivipara*), E (*Z. v. vivipara*) and F (*Z. v. vivipara*). We did not get any reliable nuclear sequences from the only two individuals of clade C (*Z. v. pannonica*) at our disposal. Analysis of these nuclear markers, thus, confirmed the monophyly of *Z. v. carniolica*. At the same time, it confirmed that the other oviparous subspecies, *Z. v. louislantzi*, is much more closely related to the viviparous subspecies. Nuclear markers, therefore, indicated a likely reversal from viviparity to oviparity, as originally hypothesised by mtDNA results [13].

## Discussion

### ESU Status

Considering not only the reproductive mode but also mtDNA and karyological features, Surget-Groba *et al*. (2002) [9] proposed to consider *Z. v. carniolica* populations from Slovenia and northwestern/northeastern Italy as an Evolutionarily Significant Unit (ESU) following Moritz (1994) [15]. According to this definition, ESU status is evaluated by taking into account both mtDNA and nuclear loci: two populations would be considered ESUs if reciprocally monophyletic at mtDNA alleles and showing significant divergence of allele frequencies at nuclear loci [15]. Although Moritz’s ESU definition has been debated (e.g. [45]), it is nonetheless widely used in the conservation field. In particular, contrasting the patterns of mtDNA and nuclear variation is routine for testing the distinctiveness of natural populations (see [46]). Due to differences in effective population size and mutation rate, nuclear DNA loci attain monophyly at a considerably slower pace than mtDNA haplotypes. Instances of concomitant monophyly at mtDNA and nuclear loci imply, therefore, a long-term history of evolutionary separation. While it is arguable whether such a separation is sufficient for the recognition of different taxonomic units (e.g., under the genealogical species concept, [47]), its importance from an evolutionary and conservation perspective cannot be neglected.

In this survey we considered all the subspecies of the *Zootoca* genus: the results showed that *Z. v. carniolica* monophyly is strongly supported by both mtDNA (cytb; Fig. 2) and nuclear markers (C-mos, ACM4 and Mc1r; Figure 5) trees. In the concatenated nuclear markers tree, the other known oviparous subspecies, *Z. v. louislantzi* (mtDNA clade B), clustered within the same clade as *Z. v. vivipara*, (mtDNA clades D, E and F) from different origins within its distribution range. We could not incorporate any individual from the *Z. v. pannonica* subspecies (mtDNA clade C) due to poor DNA quality. However, if we consider the concordance between the mitochondrial- and nuclear-based phylogeny and the previously demonstrated inclusion of *Z. v. pannonica* in the same mtDNA clade as *Z. v. vivipara* and *Z. v. louislantzi* [13], this omission would not be expected to alter the overall scenario. To the best of our knowledge, this is the first phylogenetic inference based on nuclear DNA in *Zootoca* genus. The nuclear and mitochondrial DNA tree topologies support *Z. v. carniolica* monophyly, which is also essentially confirmed by the extent of nuclear allele sharing. *Z. v. carniolica* individuals do not share any allele with individuals from other subspecies at C-mos and Mc1r genes; just two out of the five alleles of ACM4, on the other hand, are shared among individuals of *Z. v. carniolica*, *Z v. louislantzi* and *Z. v. vivipara*, most likely due to the retention of ancestral polymorphism. Finally, the concordance between mitochondrial and nuclear markers confirms the reliability of mtDNA-based discrimination of the different subspecies. This can help to assess the geographic occurrence of *Z. vivipara* subspecies that, otherwise, might be problematic if based only on morphology.

### Evolutionary, Demographic and Phylogeographical Scenarios

Adopting the mtDNA clades definition of Surget-Groba *et al*. (2006) [13], our investigation has been concentrated on clade A (eastern oviparous) and clade E (western viviparous) in a specific area (central-eastern Italian Alps) where distributions partially overlap.

The results of mtDNA and C-mos, in particular, showed much greater genetic variability in populations of *Z. v. carniolica* than in those of *Z. v. vivipara* (see Table 1), even though a far lower number of individuals of the latter have been analysed. Gene diversity was significantly higher in *carniolica* than in *vivipara* (z-test, p<0.05), in both the cytb and the C-mos.

Greater genetic diversity can be related to a longer evolutionary history (see [48]). In this case, the molecular data would confirm the phenotypic data with respect to reproductive mode, with oviparity being the ancestral condition. The reduced genetic variation of clade E (*Z. v. vivipara*) compared with clade A (*Z. v. carniolica)* could be associated to a small effective population size during the divergence from an oviparous form, followed by a more recent demographic expansion (see below) after the retreat of the ice from western central Europe.

From the phylogenetic tree (Figure 2) it emerges that oviparous clade A and viviparous clade E are not sister clades. It is, however, worth noting that the overall cytb phylogeny of the different oviparous (A and B) and viviparous (C, D, E and F) clades clearly shows that clade A is the sister clade to all the others. Our inference places the divergence of clade A at approximately 4.5 Mya (95% CI 6.1-2.6 Mya) that is during the Pliocene. This estimation should not be over-emphasized or, even worse, taken at face value. We obtained this estimated divergence time after setting, as geological calibration on the node separating *P. cretensis* from *P. peloponnesiaca*, the date the island of Crete presumably separated from the Peloponnese, according to geological evidence [29], that is 5.2 Mya before the present day. While having a more refined calibration would improve our estimate of the divergence time (see [49]), it nonetheless can be said that the sister oviparous clade A had a long evolutionary history since the original split from all the other clades of the species.

Another indication of the deep evolutionary distance between clade A and E stems from the number of differences in the cytb fragment. The average number of nucleotide differences between the two clades was 20.801+/−3.974 (hence 0.05403 per site), with a net nucleotide divergence of approximately 5% (Da = 0.049), which, for the mitochondrial cytb gene, indicates a rather large divergence. These figures are similar to observations between two distinct species belonging to a genus “close” to *Zootoca: P. peloponnesiaca* and *P. cretensis.* They showed an average number of nucleotide differences of 17.589+/−3.292 and Da of 0.047 for the same cytb marker [50].

The haplotype cytb network (Figure 3) shows another striking difference between the two subspecies: all our *Z. v. vivipara* individuals harbour haplotypes clustering in clade E, that is, according to its original definition [12], the western viviparous group. This clade is characterized by a “star-shape” topology, suggesting recent population expansion [51]. In contrast, all our *Z. v. carniolica* individuals have haplotypes belonging to clade A [12], and whose network does not present any particular topology. The indication of a demographic expansion in clade E can be further evaluated through the mismatch distribution graph (Figure 4). This graph shows a unimodal trend, which is again considered a signature of a recent demographic expansion [52]. Our distribution is not in contradiction with the theoretical model of Excoffier (2004) [52], describing an instantaneous range expansion in a two-dimensional stepping-stone model, with large migration rates and recent expansion (Figure 4, inset b). A plausible scenario would thus imply that, with the retreat of the ice in the post-Pleistocene era, populations of *Z. v. vivipara* belonging to clade E were able to spread into northwestern Europe (see [6]), leading to a simultaneous population and spatial expansion. The signature of this demographic expansion appears robust as it is also supported by the results of the neutrality tests (both Tajima’s D and Fu’s Fs, statistically significant and negative) and of the coalescent-based skyline plot [25] that shows a pattern of a relatively recent increase of Ne within clade E (Figure 4, inset a).

All the aforementioned demographic inferences are based on the assumption of neutrality for mtDNA cytb. Although the mitochondrial cytb marker was considered neutral in most of the previous studies on the phylogeography of *Z. vivipara* [9], [12], [13], a recent paper, focussed on a contact zone of two mtDNA *Z. v. louislantzi* lineages [18], questioned this assumption. The authors hypothesised a kind of thermal-related selection for explaining the differential survival of two subadults’ cytb haplogroups living in syntopy, in a secondary contact zone. It appears at least advisable to wait for new evidence (comparison of survivorship over a longer period) supporting this hypothesis. Moreover, we think that even if there is selection acting locally on mtDNA at a contact zone, it does not mean that we cannot recover a historical pattern (e.g. expansion) from other wider regions and for other lineages with allopatric distributions. Thus, we think that cytb can still be used for general demographic and phylogeographical inferences, especially if they are confirmed by other markers, like in our study.

### Biogeographical Distribution

According to our results, 149 out of 191 individuals of our Italian samples were assigned to the subspecies *Z. v. vivipara* and 42 to the subspecies *Z. v. carniolica*. This allowed clarification of the situation surrounding the distribution of the two subspecies in the 51 sites sampled. In 15 sites only the subspecies *Z. v. carniolica* was found, while in 36 sites only the subspecies *Z. v. vivipara* was present (Figure 1).

According to our study, in the central-eastern Italian Alps *Z. v. vivipara,* on average, tends to live at higher altitudes (mean 1701 m) than *Z. v. carniolica* (mean 1210 m). *Z. v. carniolica* populations can be found at higher altitudes than initially thought: at sites above 1400 m in Trentino (Tremalzo 1545 m, Lago Nero 1625 m, Palù Longa 1435 m), in Veneto (Monte Grappa 1700 m), and in Lombardy (Branzi 1800 m, Ardesio 1600 m, Roncobello 1880 m). A high altitude (1900 m) population of *Z. v. carniolica* was also identified in Piedmont (northwestern Italy) by Ghielmi *et al*. [53], [54]. *Z.v. carniolica* and *Z. v. vivipara* exhibit an indisputable overlap of their altitudinal distributions in the Italian Alps, similarly to other areas such as Carinthia, Austria, where the two subspecies have even been found in syntopy in a site at 1575 m [10].

The geographical distribution of the different haplotypes (Figure 3) corresponds to a biogeographical limit called the ‘Brenner line’ (i.e. a longitudinal line from the Adige Valley up to the Brenner Pass, Figure 1). This line has been recognized as delimiting eastern and western distributions of many plant species since the 19^th^ century ([55] and see other examples below). All populations of *Z. v. vivipara* on the east of this line have VB11 (or derived haplotypes), while all population on the west have VB1 (or derived haplotypes). The only exception is one sample in population 12, which is on the west but shows VB11. The same pattern of east-west division by the Brenner line seems to hold for our *Z. v. carniolica* haplotypes of clade A. In this case, haplotypes OS8 and OT_11 belong only to individuals from sites east of this line. These two haplotypes cluster together with OS9, a haplotype described by Surget-Groba *et al.,* (2006) [13] and found in individuals from the Italian province of Udine that is located far east of the Brenner line.

The nuclear marker phylogenetic tree does not present the same biogeographical pattern: it is likely that the slow mutation rate of these markers limits their phylogeographical informativeness.

Further research with markers better suited for fine-scale population genetics analyses, such as microsatellites, could confirm this preliminary indication of a possible east-west differentiation along the Brenner line. This pattern would be in line with what have been already found in the high-altitude butterfly, *Erebia euryale* [56], in an Alpine form of rampion, *Phyteuma globulariifolium,* in the alpine speedwell, *Veronica alpina,* [57], [58] and in many other plant species [59], [60].

### Implications for Conservation

Considering the evidence from karyotype to cytb variation, and the results of our study, the distinction between *Z. v. vivipara* and *Z. v. carniolica* can be regarded as evolutionarily substantial. While it is arguable whether such distinction deserves a taxonomical revision, we think that nonetheless it has some important consequences for conservation. In our view, proposing specific conservation action for *Z. v. carniolica* is further strengthened by a number of important aspects.

The low and medium altitude peatland habitats that *Z. v. carniolica* prefers are already thought to be at high risk of extinction [61]. Indeed, the European Habitats Directive 43/92/CEE has classified active raised bogs (Natura Code 2000: 7110), transition and quaking bogs (7140), and alkaline fens (7230) as either threatened (7140, 7230) or even seriously threatened (7110). In particular, peatlands across the Alps are suffering from a reduction in both the surface area of individual peatlands and their total number.

Moreover, a study on the peat bogs of Italian Alps [62] revealed that heat waves, like that of 2003, affected the survival of organisms such as peat mosses (genus *Sphagnum*), which play a crucial role in maintaining bog functionality (i.e. carbon storage). Such a drastic change in mountain peat bogs due to just a single summer of higher temperatures and reduced rainfall represents a major concern with respect to the conservation status of this habitat. Exceptionally hot European summers, like that of 2003, may occur more frequently given recent climatic changes, bringing perilous consequences for mountain peatlands and their associated flora and fauna like *Z. v. carniolica*.

## Conclusions

The main conclusion of our study is that the reciprocal monophyly between the oviparous subspecies *Z. v. carniolica* and all the other *Z. vivipara* subspecies has been proved for the first time using nuclear DNA markers This now makes it possible to properly consider *Z. v. carniolica* as an ESU. The macro- and micro-scale analysis of the evolutionary history of *Z. v. carniolica* allowed us to reach the following conclusions: i) according to an external fossil calibration, the divergence time between *Z. v. carniolica* and all the other subspecies took place at least 2.6 millions years before the present day, thus corresponding to a relatively long time of evolutionary separation; ii) also in terms of demographic history, there is a remarkable difference: *Z. v. carniolica* does not show any signature of expansion as it occurs in the most widespread *Z. v. vivipara* clade (clade E of central-northern Europe); iii) the genetic evidence of this study, together with the vulnerability of *Z. v. carniolica* most suitable habitats (i.e. low-mid altitudes peat bog), suggests specific action tailored to this subspecies.

While future studies could better address the recent findings of sintopy and of possible hybridization between *Z. v. carniolica* and *Z. v. vivipara* [10], a clear evolutionarily and demographic distinction has now been demonstrated, much likely prompting a taxonomical revision.

## Supporting Information

Figure S1 **Distribution of** ***Zootoca*** **subspecies.** Highlighted rectangle represents the area of the study.
(TIF)

Figure S2 **Median joining network of three nuclear genes.** Circles represent phased alleles, area is proportional to frequency and colour indicates the mtDNA clade (see legend).
(TIF)

Figure S3 **Maximum clade credibility trees of Bayesian analyses of three nuclear genes.** Bootstrap support values of Maximum Likelihood analysis >70% are shown above the branches, while posterior probability values of Bayesian Inference >0.7 are shown below the branches. *Atlantolacerta andreanskyi* was used as outgroup.
(TIF)

Figure S4 ***Z. v. carniolica*** **(Clade A) cyt b mismatch distribution.** The number of nucleotide site differences between pair of individuals and the frequency of observation, are reported on the x- and y-axis respectively. Dashed and thick lines represent observed and expected (under sudden expansion model) distribution, respectively. In the inset a) the Bayesian skyline plot and 95% Credibility Interval.
(TIF)

Table S1 **Tables S1a and S1b. a. (Supporting** **Figure 1** **) Sampling sites details across Italian Alps.** Number of samples collected for each site (N), GPS Coordinates, Altitude and mtDNA cyt b haplotype and alleles observed for each nuclear gene. Numbers in brackets refer to allele frequencies. **Table S1b. (Supporting Figure S1) Sampling sites details across Europe.** Number of samples for each site (N), Country, subspecies, mtDNA clade and alleles observed for each nuclear gene. Numbers in brackets refer to haplotypes (mtDNA) and allele frequencies (nuclear genes). * Heulin et al. (2011) § Surget-Groba et al. (2006).
(PDF)

Table S2 **Sequence accession numbers.** MtDNA haplotypes and phased nuclear alleles sequences found in this study and their relative database accession number.
(PDF)
