# Supplementary figures and images for: Mitochondrial and Nuclear DNA Survey of Zootoca vivipara across the Eastern Italian Alps: Evolutionary Relationships, Historical Demography and Conservation Implications
Source: PLoS One. 2014 Jan 17;9(1):e85912. doi: 10.1371/journal.pone.0085912 (PMC3895026; doi:10.1371/journal.pone.0085912)

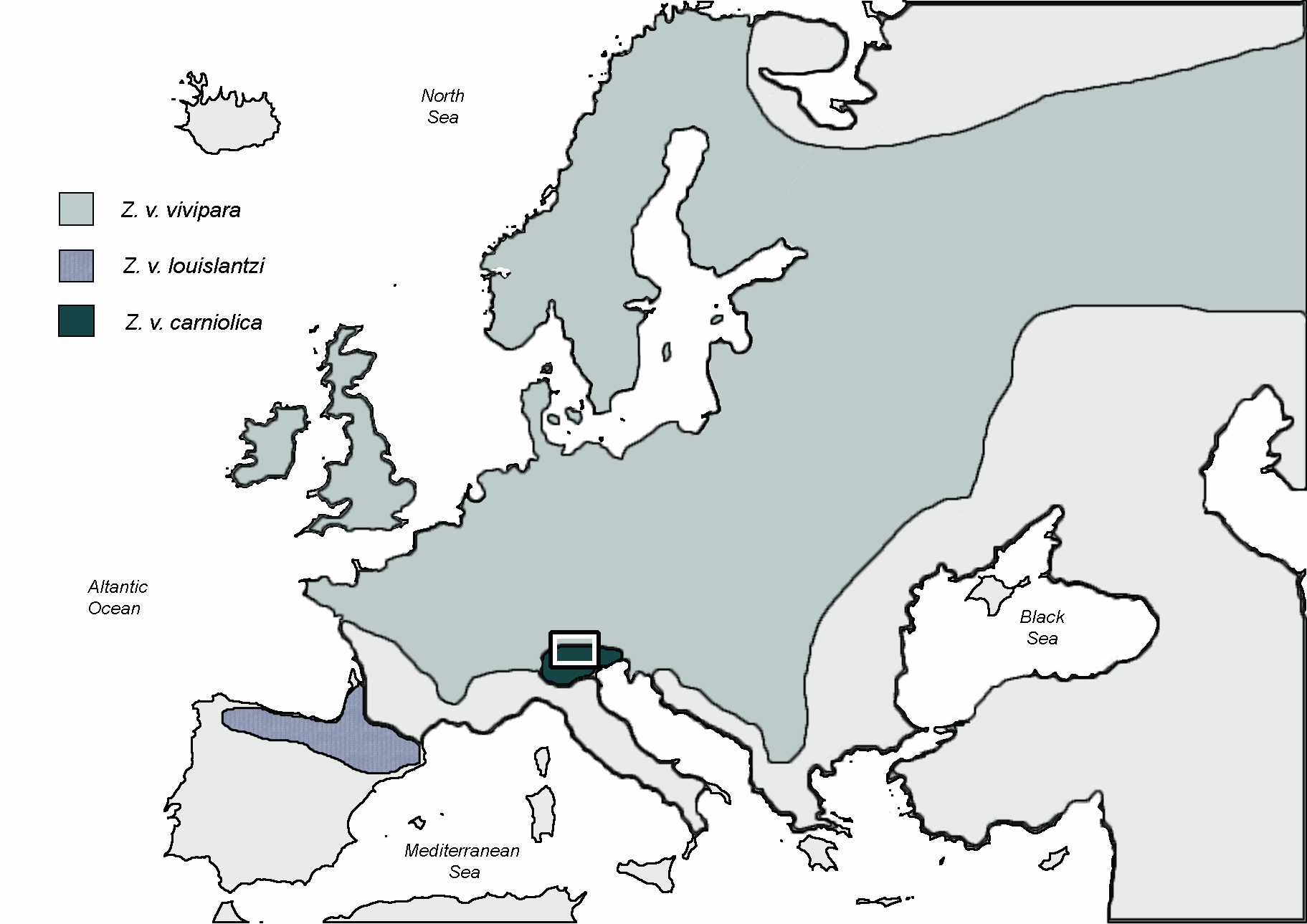

Supplement: Figure S1 — Distribution of Zootoca subspecies. Highlighted rectangle represents the area of the study. (TIF) [file pone.0085912.s001.tif]

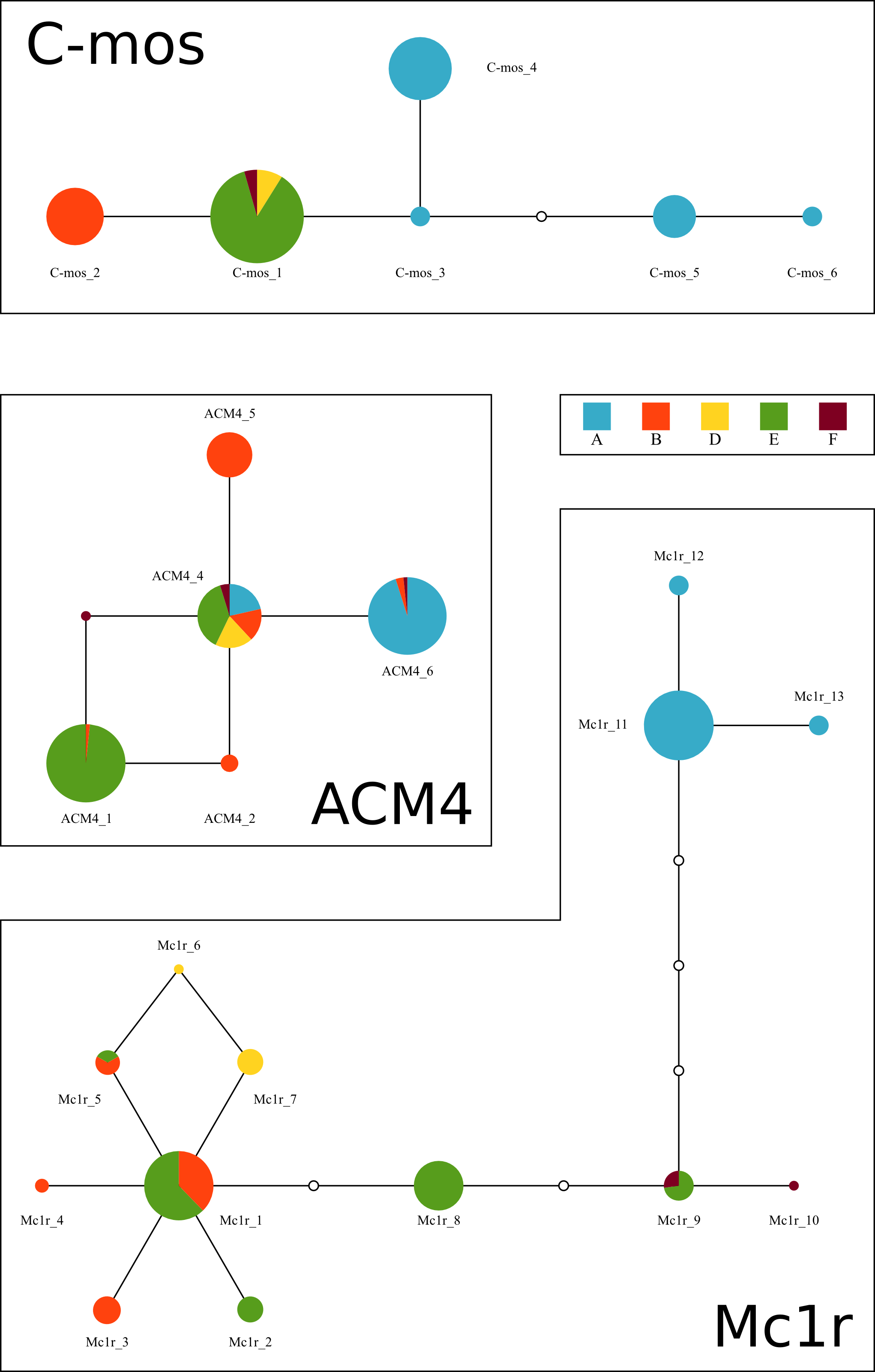

Supplement: Figure S2 — Median joining network of three nuclear genes. Circles represent phased alleles, area is proportional to frequency and colour indicates the mtDNA clade (see legend). (TIF) [file pone.0085912.s002.tif]

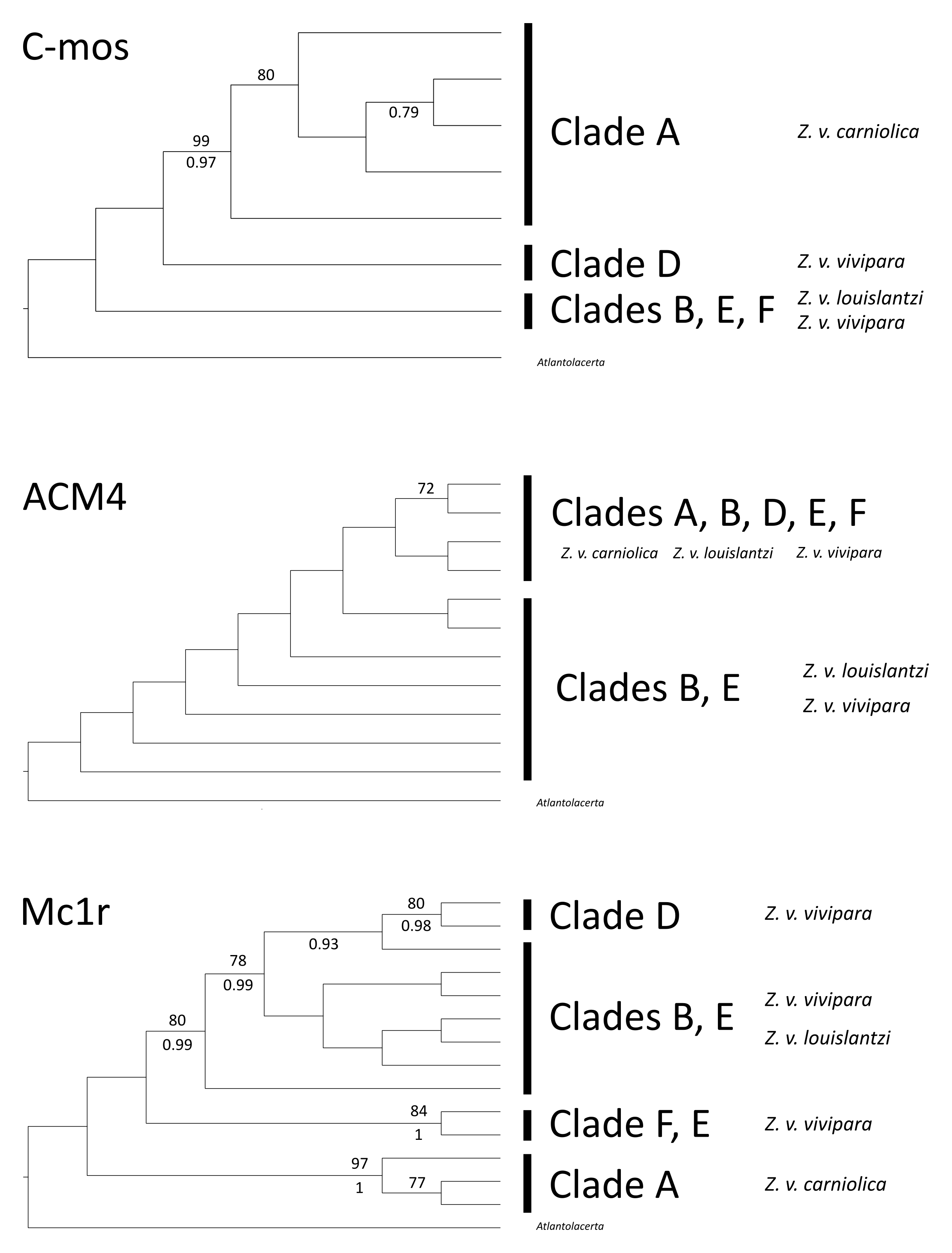

Supplement: Figure S3 — Maximum clade credibility trees of Bayesian analyses of three nuclear genes. Bootstrap support values of Maximum Likelihood analysis >70% are shown above the branches, while posterior probability values of Bayesian Inference >0.7 are shown below the branches. Atlantolacerta andreanskyi was used as outgroup. (TIF) [file pone.0085912.s003.tif]

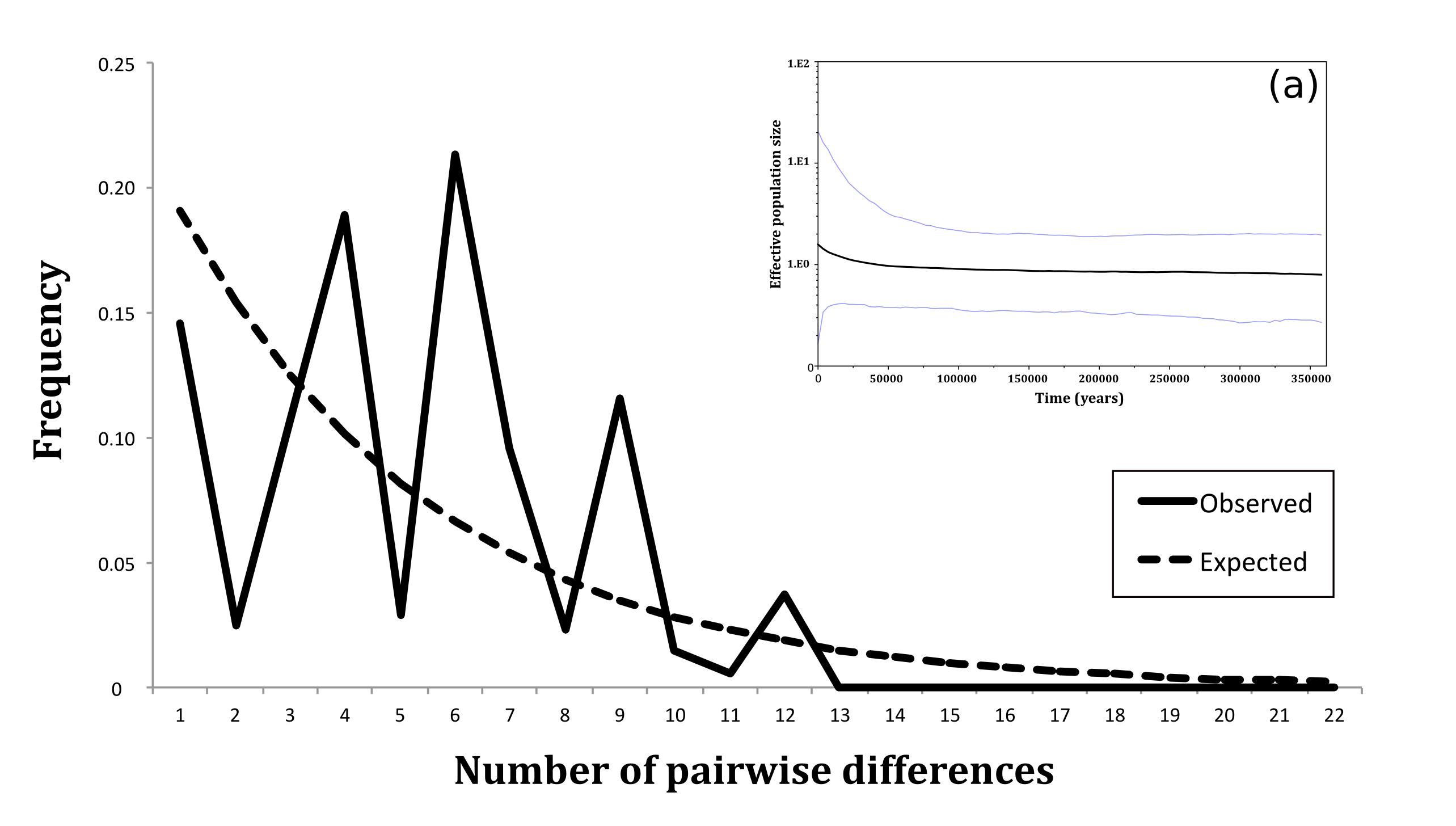

Supplement: Figure S4 — Z. v. carniolica (Clade A) cyt b mismatch distribution. The number of nucleotide site differences between pair of individuals and the frequency of observation, are reported on the x- and y-axis respectively. Dashed and thick lines represent observed and expected (under sudden expansion model) distribution, respectively. In the inset a) the Bayesian skyline plot and 95% Credibility Interval. (TIF) [file pone.0085912.s004.tif]
