# Supplementary material for: Mitochondrial and Nuclear DNA Survey of Zootoca vivipara across the Eastern Italian Alps: Evolutionary Relationships, Historical Demography and Conservation Implications
Source: PLoS One. 2014 Jan 17;9(1):e85912. doi: 10.1371/journal.pone.0085912 (PMC3895026; doi:10.1371/journal.pone.0085912)
Supplement: Table S1 — Tables S1a and S1b. a. (Supporting Figure 1 ) Sampling sites details across Italian Alps. Number of samples collected for each site (N), GPS Coordinates, Altitude and mtDNA cyt b haplotype and alleles observed for each nuclear gene. Numbers in brackets refer to allele frequencies. Table S1b. (Supporting Figure S1) Sampling sites details across Europe. Number of samples for each site (N), Country, subspecies, mtDNA clade and alleles observed for each nuclear gene. Numbers in brackets refer to haplotypes (mtDNA) and allele frequencies (nuclear genes). * Heulin et al. (2011) § Surget-Groba et al. (2006). (PDF) [file pone.0085912.s005.pdf]

Table S1a.

	Sites	N	GPS coordinates North	East	Altitude (m asl)	mtDNA clade	cyt b haplotypes	Cmos	ACM4	Mc1r
1	P. Lavazè (Peat bog)	11	46°20'25"	11°29'13"	1565	E	VB11(11)	C-mos_1(2)	ACM4_4(2)	Mc1r_9(2)
2	P. Lavazè (Lake)	5	46°21'22"	11°29'33"	1805	E	VB11(5)	C-mos_1(2)	ACM4_1(2)	Mc1r_8(2)
3	Costa	3	45°54'42"	11°11'38"	1250	A	OS8(3)	C-mos_4(2)	ACM4_6(1), ACM4_4(1)	Mc1r_11(2)
4	Passo Manghen	21	46°10'39"	11°27'12"	2060	E	VB11(2), VT_24(1), VB1(18)	C-mos_1(8)	ACM4_1(5), ACM4_4(3)	Mc1r_1(4), Mc1r_2(4)
5	Lago d' Ampola	13	45°52'19"	10°39'16"	735	A	OS6(13)	C-mos_6(4), C-mos_5(2), C-mos_4(14)	ACM4_6(18), ACM4_4(2)	Mc1r_12(1), Mc1r_11(15), Mc1r_13(4)
6	Passo San Pellegrino	4	46°22'29"	11°45'32"	1795	E	VB11(4)	C-mos_1(4)	ACM4_1(4)	Mc1r_1(4)
7	Palù Longa	12	46°17'41"	11°21'58"	1435	A	OT_11(2), OS8(10)	C-mos_4(20)	ACM4_6,ACM4_4	Mc1r_11(20)
8	Passo Tonale	10	46°15'31"	10°35'52"	1850	E	VB1(9), VT_23(1)	C-mos_1(12)	ACM4_1(12)	Mc1r_8(4), Mc1r_1(5), Mc1r_2(3)
9	Masi Carretta	12	46°06'24"	11°37'51"	1305	E	VB1(2), VB11(10)	C-mos_1(2)	ACM4_1(2)	Mc1r_8(2)
10	Passo Redebus	3	46°08'20"	11°19'04"	1435	E	VB1(2), VB11(1)	NA	NA	NA
11	Palù Longia	6	46°28'20"	11°04'58"	1565	E	VB1(6)	C-mos_1(2)	ACM4_1(2)	Mc1r_5(2)
12	Palù Tremole	14	46°28'44"	11°04'30"	1720	E	VB1(13), VB11(1)	C-mos_1(2)	ACM4_1(2)	Mc1r_8(2)
13	Lago Calaita	7	46°12'21"	11°47'38"	1660	E	VB1(6), VB11(1)	NA	NA	NA
14	Biotopo "I Mughi"	5	46°05'52"	11°36'38"	1220	E	VB1(3),VT_24(2)	NA	NA	NA
15	Passo Brocon	1	46°07'13"	11°41'10"	1670	E	VB11(1)	NA	NA	NA
16	Malga Ces, Siror	1	46°16'17"	11°46'19"	1680	E	VB11(1)	NA	NA	NA
17	Passo Valles	1	46°20'22"	11°47'58"	2030	E	VB11(1)	NA	NA	NA
18	Soraga	2	46°23'25"	11°40'00"	1205	E	VB11(2)	NA	NA	NA
19	Inghiaie	1	45°59'51"	11°18'37"	450	A	OS8(1)	NA	NA	NA
20	Laghi di Colbricon	3	46°16'59"	11°45'59"	1915	E	VB11(3)	C-mos_1(2)	ACM4_1(2)	Mc1r_2(2)
21	Campo Carlo Magno	5	46°15'30"	10°50'50"	1650	E	VB1(5)	NA	NA	NA
22	Lago Nero	1	46°16'53"	11°19'39"	1625	A	OS8(1)	NA	NA	NA
23	Laghetti di Lasteati	6	46°10'04"	11°33'30"	2080	E	VB1(4), VB11(2)	NA	NA	NA
24	Croda Rossa	2	46°13'01"	10°53'06"	2160	E	VB1(2)	NA	NA	NA
25	Val de la Mare	2	46°23'32"	10°41'29"	1400	E	VB1(2)	NA	NA	NA

26	Tremalzo	1	45°50'25"	10°40'56"	1545	A	OS6(1)	C-mos_4(2)	ACM4_6(2)	Mc1r_11(2)
27	Valle Vernecolo	2	46°02'36"	10°09'26"	1800	E	VB1(2)	C-mos_1(2)	ACM4_1(2)	Mc1r_8(2)
28	Malga Lifretto	1	46°01'05"	10°12'58"	1400	E	VB1(1)	NA	NA	NA
29	Campelli	1	46°00'45"	10°10'40"	1160	E	VB1(1)	C-mos_1(2)	ACM4_1(2)	Mc1r_8(2)
30	Valle del Vò	2	46°02'57"	10°07'46"	1810	E	VB1(2)	C-mos_1(2)	ACM4_1(2)	Mc1r_8(2)
31	Branzi	1	46°00'10"	9°47'06"	1800	A	OS3(1)	C-mos_5(2)	ACM4_6(2)	Mc1r_11(2)
32	Mezzoldo	1	46°02'33"	9°37'03"	1800	E	VB1(1)	C-mos_1(2)	ACM4_1(2)	Mc1r_8(2)
33	Averara	1	46°01'36"	9°37'43"	1415	A	OS3(1)	C-mos_3(2)	ACM4_6(1), ACM4_4(1)	Mc1r_11(1), Mc1r_13(1)
34	Cusio	1	45°59'02"	9°36'06"	1125	A	OS3(1)	C-mos_5(2)	ACM4_6(1), ACM4_4(1)	Mc1r_11(1), Mc1r_13(1)
35	Vilminore	5	46°00'35"	10°01'55"	1640	E	VB1(5)	C-mos_1(2)	ACM4_1(2)	Mc1r_8(2)
36	Ornica	1	45°59'54"	9°33'39"	1330	A	OS3(1)	C-mos_5(2)	ACM4_6(2)	Mc1r_11(2)
37	Valgoglio	2	45°57'58"	9°52'06"	1420	A	OL_11(2)	C-mos_5(2)	ACM4_6(2)	Mc1r_11(2)
38	Ardesio	1	45°52'00"	9°50'57"	1600	A	OL_11(1)	C-mos_5(2)	ACM4_6(2)	Mc1r_11(2)
39	Val Bondione	3	46°01'21"	10°00'39"	1290	E	VB1(3)	C-mos_1(4)	ACM4_1(4)	Mc1r_1(4)
40	Oneta	1	45°51'49"	9°47'37"	1320	A	OL_12(1)	C-mos_4(2)	ACM4_6(2)	Mc1r_11(2)
41	Valleve	1	46°03'06"	9°41'49"	1830	E	VB1(1)	C-mos_1(2)	ACM4_1(2)	Mc1r_11(2)
42	Gandellino	4	46°00'18"	9°54'43"	1720	E	VB1(4)	C-mos_1(4)	ACM4_1(4)	Mc1r_8(2), Mc1r_1(2)
43	Roncobello	1	45°57'55"	9°47'30"	1880	A	OL_11(1)	C-mos_3(2)	ACM4_6(2)	Mc1r_11(2)
44	Ridanna	1	46°56'30"	11°15'00"	1715	E	VB1(1)	C-mos_1(2)	ACM4_1(2)	Mc1r_1(2)
45	Vipiteno	1	46°56'50"	11°18'00"	1815	E	VB1(1)	C-mos_1(2)	ACM4_1(2)	Mc1r_1(2)
46	Sesto	1	46°39'43"	12°21'07"	1470	E	VB11(1)	C-mos_1(2)	ACM4_4(2)	Mc1r_9(2)
47	Campo Tures	1	46°58'47"	12°05'39"	1860	E	VB11(1)	C-mos_1(2)	ACM4_4(1), ACM4_1(1)	Mc1r_1(2)
48	Redagno	2	46°20'41"	11°23'55"	1540	E	VB11(2)	NA	NA	NA
49	Dobbiaco	1	46°42'10"	12°13'17"	1250	E	VB11(1)	C-mos_1(2)	ACM4_4(2)	Mc1r_1(2)
50	Monte Grappa	2	45°52'20"	11°18'09"	1700	A	OS8(2)	NA	NA	NA
51	Sappada	1	46°37'15"	12°42'49"	1830	E	VB11(1)	C-mos_1(2)	ACM4_4(2)	Mc1r_9(2)
	**Totale**	**191**					**191**			

Table S1b.

Sites	N	Country	Subspecies	mtDNA clade	Cmos	ACM4	Mc1r
Pinet-Bélesta	1	France	*Z. v. louislantzi*	B*	C-mos_2 (2)	ACM4_4 (2)	Mc1r_1 (2)
Clamondé	1	France	*Z. v. louislantzi*	B*	C-mos_2 (2)	ACM4_2 (2)	Mc1r_1 (2)
Etang de Lers	1	France	*Z. v. louislantzi*	B*	C-mos_2 (2)	ACM4_4 (2)	Mc1r_1 (2)
Clarens	1	France	*Z. v. louislantzi*	B*	C-mos_2 (2)	ACM4_5 (1), ACM4_1 (1)	Mc1r_5 (2)
Louvie	2	France	*Z. v. louislantzi*	B*	C-mos_2 (4)	ACM4_5 (4)	Mc1r_1 (2), Mc1r_5 (2)
Pourtalet	2	Spain	*Z. v. louislantzi*	B*	C-mos_2 (4)	ACM4_5 (4)	Mc1r_1, (2) Mc1r_3 (2)
Iraty	1	France	*Z. v. louislantzi*	B*	C-mos_2 (2)	ACM4_4 (2)	Mc1r_3 (2)
La Rhune	1	France	*Z. v. louislantzi*	B*	C-mos_2 (2)	ACM4_5 (2)	Mc1r_3 (2)
Gabas	7	France	*Z. v. louislantzi*	B*	C-mos_2 (14)	ACM4_5 (10), ACM4_4 (1), ACM4_6 (2), ACM4_2 (1)	Mc1r_4 (2), Mc1r_1 (10), Mc1r_3 (2)
Szklarska Poreba	1	Poland	*Z. v. vivipara*	E§	C-mos_1 (2)	ACM4_1 (2)	Mc1r_1 (2)
Ustrzyki Gorne	1	Poland	*Z. v. vivipara*	E§	C-mos_1 (2)	ACM4_4 (2)	Mc1r_9 (2)
Paimpont	1	France	*Z. v. vivipara*	E§	C-mos_1 (2)	ACM4_1 (2)	Mc1r_8 (2)
Tarpa	1	Hungary	*Z. v. vivipara*	E§	C-mos_1 (2)	ACM4_4 (2)	Mc1r_1 (2)
Krutyn	1	Bulgaria	*Z. v. vivipara*	E§	C-mos_1 (2)	ACM4_1 (2)	Mc1r_8 (2)
Turukchanskii Krai	1	Russia	*Z. v. vivipara*	D§	C-mos_1 (2)	ACM4_4 (2)	Mc1r_7 (1), Mc1r_6 (1)
Sakhaline	1	Russia	*Z. v. vivipara*	D§	C-mos_1 (2)	ACM4_4 (2)	Mc1r_7 (2)
Grossevitchi	1	Russia	*Z. v. vivipara*	D§	C-mos_1 (2)	ACM4_4 (2)	Mc1r_7 (2)
Kara-Khol	1	Russia	*Z. v. vivipara*	D§	C-mos_1 (2)	ACM4_4 (2)	Mc1r_7 (2)
Godingberg	1	Austria	*Z. v. vivipara*	F§	C-mos_1 (2)	ACM4_4 (1), ACM4_6 (1)	Mc1r_9 (2)
Emberger Alm	1	Austria	*Z. v. vivipara*	F§	C-mos_1 (2)	ACM4_3 (1), ACM4_4 (1)	Mc1r_9 (1), Mc1r_10 (1)
