# Supplementary material for: Mitochondrial and Nuclear DNA Survey of Zootoca vivipara across the Eastern Italian Alps: Evolutionary Relationships, Historical Demography and Conservation Implications
Source: PLoS One. 2014 Jan 17;9(1):e85912. doi: 10.1371/journal.pone.0085912 (PMC3895026; doi:10.1371/journal.pone.0085912)
Supplement: Table S2 — Sequence accession numbers. MtDNA haplotypes and phased nuclear alleles sequences found in this study and their relative database accession number. (PDF) [file pone.0085912.s006.pdf]

	Sequence	Accession number
Phased nuclear alleles	C-mos_1	EF632292
	C-mos_2	KF886547
	C-mos_3	KF886546
	C-mos_4	KF886545
	C-mos_5	KF886543
	C-mos_6	KF886544
	ACM4_1	KF886565
	ACM4_2	KF886564
	ACM4_3	KF886566
	ACM4_4	KF886563
	ACM4_5	KF886562
	ACM4_6	KF886561
	Mc1r_1	KF886555
	Mc1r_2	KF886556
	Mc1r_3	KF886557
	Mc1r_4	KF886558
	Mc1r_5	KF886552
	Mc1r_6	KF886554
	Mc1r_7	KF886553
	Mc1r_8	KF886559
	Mc1r_9	KF886550
	Mc1r_10	KF886551
	Mc1r_11	KF886548
	Mc1r_12	KF886549
	Mc1r_13	KF886560
MtDNA haplotypes	VB11	AY714892
	VB1	AY714882
	VT_23	KF886538
	VT_24	KF886539
	OS3	AY714923
	OS6	AF444041
	OS8	AY714927
	OT_11	KF886540
	OL_11	KF886541
	OL_12	KF886542
